# Stress sensing in plants by an ER stress sensor/transducer, bZIP28

**DOI:** 10.3389/fpls.2014.00059

**Published:** 2014-02-26

**Authors:** Renu Srivastava, Yan Deng, Stephen H. Howell

**Affiliations:** ^1^Plant Sciences Institute, Iowa State UniversityAmes, IA, USA; ^2^Department of Genetics, Development and Cell Biology, Iowa State UniversityAmes, IA, USA

**Keywords:** endoplasmic reticulum stress, unfolded protein response (UPR), bZIP transcription factors, binding immunoglobulin protein (BIP), protein folding, Golgi apparatus, COPII vesicle transport system

## Abstract

Two classes of ER stress sensors are known in plants, membrane-associated basic leucine zipper (bZIP) transcription factors and RNA splicing factors. ER stress occurs under adverse environmental conditions and results from the accumulation of misfolded or unfolded proteins in the ER lumen. One of the membrane-associated transcription factors activated by heat and ER stress agents is bZIP28. In its inactive form, bZIP28 is a type II membrane protein with a single pass transmembrane domain, residing in the ER. bZIP28’s N-terminus, containing a transcriptional activation domain, is oriented towards the cytoplasm and its C-terminal tail is inserted into the ER lumen. In response to stress, bZIP28 exits the ER and moves to the Golgi where it is proteolytically processed, liberating its cytosolic component which relocates to the nucleus to upregulate stress-response genes. bZIP28 is thought to sense stress through its interaction with the major ER chaperone, binding immunoglobulin protein (BIP). Under unstressed conditions, BIP binds to intrinsically disordered regions in bZIP28’s lumen-facing tail and retains it in the ER. A truncated form of bZIP28, without its C-terminal tail is not retained in the ER but migrates constitutively to the nucleus. Upon stress, BIP releases bZIP28 allowing it to exit the ER. One model to account for the release of bZIP28 by BIP is that BIP is competed away from bZIP28 by the accumulation of misfolded proteins in the ER. However, other forces such as changes in energy charge levels, redox conditions or interaction with DNAJ proteins may also promote release of bZIP28 from BIP. Movement of bZIP28 from the ER to the Golgi is assisted by the interaction of elements of the COPII machinery with the cytoplasmic domain of bZIP28. Thus, the mobilization of bZIP28 in response to stress involves the dissociation of factors that retain it in the ER and the association of factors that mediate its further organelle-to-organelle movement.

## INTRODUCTION

The endoplasmic reticulum (ER) engages in the folding and modification of proteins in the endomembrane system to ensure their correct sorting, secretion and function. Disturbances in the ER or overload in secreted protein production results in the accumulation of unfolded proteins, which has the potential to damage cells. This condition is sensed by specialized stress sensors/transducers in the ER membrane, which elicit the unfolded protein response (UPR). Plants have two kinds of sensor/transducers, ER membrane-associated basic leucine zipper (bZIP) transcription factors and RNA splicing factors. Upon stress, these sensors/transducers initiate several cellular responses that transduce signals to the nucleus to help restore ER homeostasis. One of the stress sensor/transducers in *Arabidopsis* is a membrane-associated transcription factor called bZIP28 that is activated by heat and ER stress agents. In this review, the mechanisms involved in stress sensing and mobilization of bZIP28 are discussed.

### STRUCTURE OF bZIP28

**FIGURE 1:**
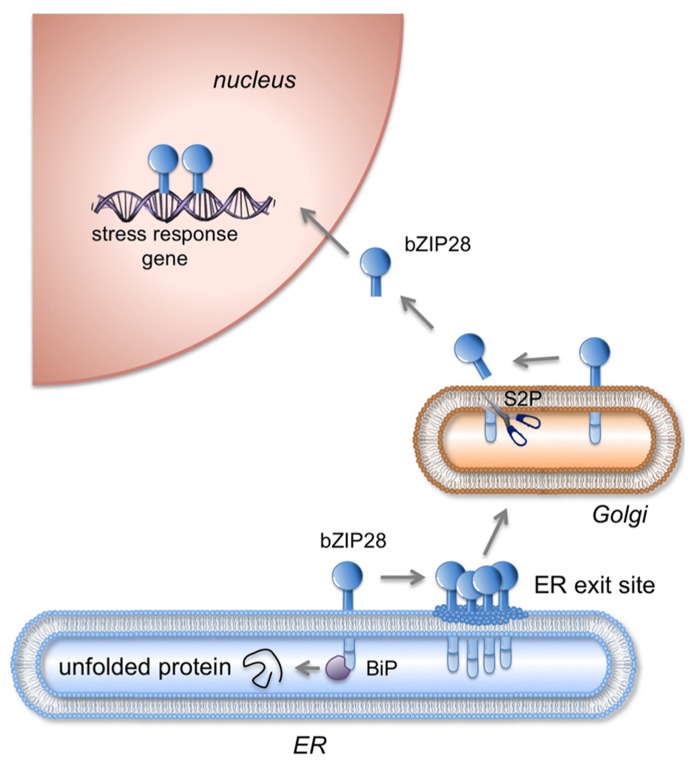
** Mobilization of bZIP28 in response to stress**. Under unstressed conditions, bZIP28 resides in the ER membrane and is thought to be tethered there by the interaction of its lumenal tail with Binding Protein (BIP). In response to adverse environmental conditions or to an overload in the protein synthesis, unfolded proteins accumulate in the ER and BIP is competed away from its binding to bZIP28. Once freed, bZIP28 interacts with Sar1 GTPase a component of the COPII transport system and transported to the Golgi apparatus. In the Golgi, bZIP28 is processed by resident proteases including Site-2-Protease (S2P) liberating bZIP28’s cytoplasmic domain, which relocates in the nucleus where it upregulates stress response genes. (Figure based on Srivastava et al., 2013).

**FIGURE 2:**
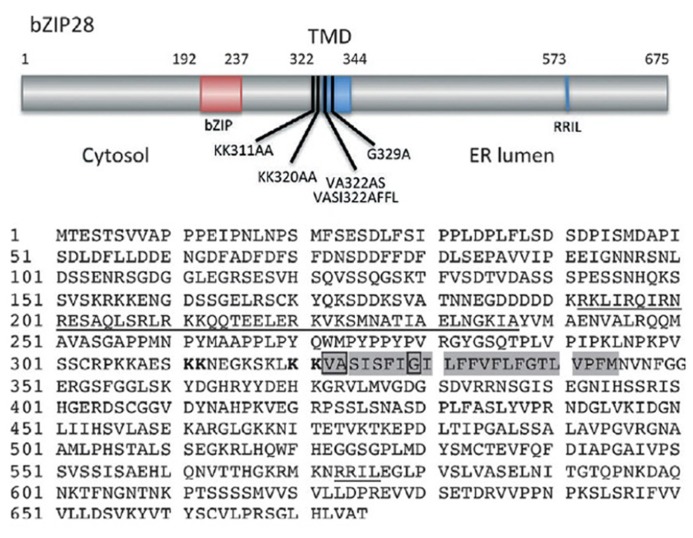
** Map (above) of *Arabidopsis* bZIP28 showing its cytosolic, transmembrane (TMD) and lumenal domains**. Also shown are the locations of the bZIP region in the cytosolic domain, the canonical S1P recognition site (RRIL) in the lumenal domain, several alanine substitution mutations and a deletion mutation (bZIP28Δ355) described in the text. Amino acid sequence of bZIP28 (below) highlights the bZIP domain and S1P recognition site (both underlined), the TMD in which the VASI sequence and helix-breaking G residue are boxed. Paired lysines involved in Sar1b binding and Golgi relocalization are indicated in bold. (Figure based on Srivastava et al., 2012).

bZIP28 is a type II membrane protein with a single pass transmembrane domain (TMD) that resides in the ER under unstressed conditions and in response to stress relocates to the nucleus where it upregulates stress response genes (**Figure 1**). bZIP28’s N-terminus contains a bZIP domain and is oriented towards the cytoplasm. The C-terminus of the protein is inserted into the ER lumen and constitutes the lumenal domain (LD) which contains a Site 1 Protease (S1P) processing site (Liu et al., 2007) and a Site 2 Protease (S2P) recognition site, which is present within the TMD (**Figure 2**). Proximal to the TMD on the cytoplasmic side are present paired lysine residues that play an important role in the translocation of bZIP28 (Srivastava et al., 2012).

### SENSING OF STRESS AND ACTIVATION OF bZIP28

Sensing of adverse environmental conditions is critical to bZIP28’s function and in its ability to protect plants from stress. bZIP28 is a key player in UPR because it is activated by ER stress and directly targets typical UPR genes (Liu et al., 2007; Gao et al., 2008; Liu and Howell, 2010b; Iwata and Koizumi, 2012; Howell, 2013). Upon stress, bZIP28 exits from the ER and moves to the Golgi where it is proteolytically processed in a sequential manner by S1P and S2P (**Figure 1**) liberating its cytosolic component (Liu et al., 2007; Che et al., 2010). The cytosolic component containing the bZIP DNA binding and dimerization domain then relocates to the nucleus, and via recruitment of NF-Y subunits upregulates stress-response genes (Liu et al., 2007; Liu and Howell, 2010a)

### ROLE OF BINDING IMMUNOGLOBULIN PROTEIN

As a sensor/transducer of UPR in plants, bZIP28 is thought to respond to ER stress in a manner similar to ATF6 in mammalian cells (Haze et al., 1999). ATF6 is also an ER membrane-bound bZIP transcription factor, with a sensor element located in the ER lumen (Chen et al., 2002).

bZIP28 senses stress through its LD (Srivastava et al., 2013; Sun et al., 2013) and through its interaction with the major ER chaperone, BIP, also known as the 78kDa glucose-regulated protein (GRP-78), which is located in the lumen of the ER (Ting and Lee, 1988; Hendershot et al., 1994; Hendershot, 2004). BIP binds to the newly synthesized proteins as they are translocated into the ER and assists in their proper folding and assembly.

**FIGURE 3:**
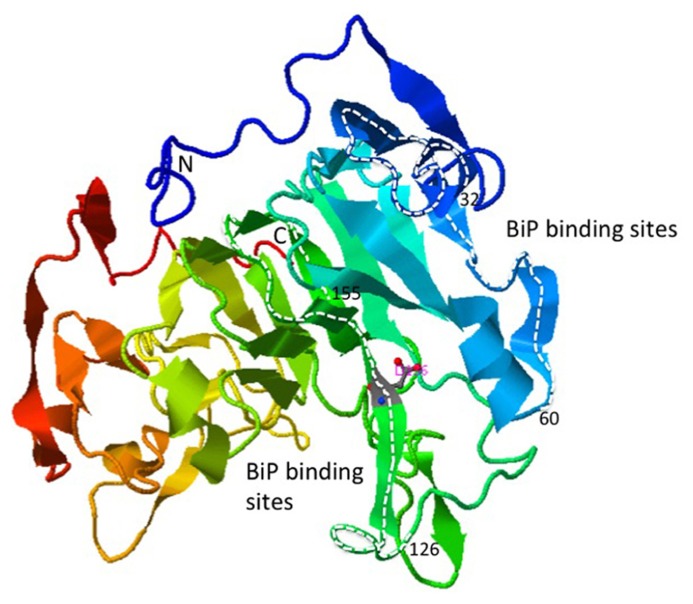
** Ribbon structure of the bZIP28 lumenal domain predicted by the template-based prediction program, I-TASSER** (Zhang, 2008). The regions of the protein highlighted with the white dashed lines represent the peptides to which BIP preferentially binds in a phage display assay (Srivastava et al., 2013).

BIP binds to bZIP28 under unstressed conditions preventing its mobilization in the absence of stress. It is not clear how the binding of BIP prevents bZIP28 mobilization. One idea derived from the mammalian literature is that BIP occludes the Golgi targeting signals on ATF6 (Shen et al., 2002). In *Arabidopsis*, BIP binds to the intrinsically disordered regions on bZIP28’s lumen-facing tail (Srivastava et al., 2013), and it is not known whether that binding interferes with cargo recognition sites needed for bZIP28’s transport from the ER to the Golgi. The crystal structure of the LD of bZIP28 has not been determined, but the predicted structure consists of a prominent β-barrel with two internal projections containing α-helix and random-coil regions (**Figure 3**). It is to the internal projections that BIP most avidly binds. In any case, BIP is released from bZIP28 in response to ER stress enabling it to exit from the ER.

The proposition that bZIP28’s lumen-facing tail has Golgi localization signals (GLSs) is not in keeping with the observation that when its tail is eliminated, the protein is not retained in the ER but behaves as an activated form of bZIP28 (Srivastava et al., 2013; Sun et al., 2013). In the study by Srivastava et al. (2013), bZIP28Δ355 constitutively relocates to the nucleus where it upregulates stress response genes, such as *BiP3*. The movement of bZIP28 takes place via the Golgi and requires S2P processing. This was demonstrated by the fact that in an S2P mutant, bZIP28Δ355 does not move into the nucleus. It is interesting to note that bZIP28Δ355 lacks a S1P processing site. Cleavage at the S1P site is usually considered to be a prerequisite for S2P cleavage, which releases the transcriptional component of stress sensor/transducer from the Golgi for relocation to the nucleus (Espenshade et al., 1999; Shen and Prywes, 2004). This implies that S1P cleavage is not required for S2P proteolysis as long as the C-terminal tail of bZIP28 has been removed.

A model to account for the release of bZIP28 by BIP under stress is a dynamic competition model in which BIP is competed away from bZIP28 by the accumulation of misfolded proteins in the ER. The model was developed in mammalian cells to explain the activation of ATF6 and IRE1 by the dissociation of BIP (Bertolotti et al., 2000; Shen et al., 2005). There are different ideas as to how BIP relinquishes its hold on ATF6 under stress conditions (Harding et al., 2002; Kaufman et al., 2002; Kimata et al., 2003; Parmar and Schroder, 2012). BIP bound to ATF6 is thought to be in equilibrium with free BIP and the BIP associated with the misfolded proteins. When unfolded proteins accumulate in the ER as a result of stress, the binding of BIP to ATF6 is competed away. Support for this model in mammalian cells has come from the overexpression of BIP, which was shown to attenuate UPR (Dorner et al., 1992; Kohno, 2010). The effects of BIP underexpression have been difficult to document in mammalian cells, which have only one BIP, and its knockout is lethal (Shen et al., 2002).

*Arabidopsis* has three BIP genes (*BIP1*, -*2*, and -*3*). BIP1 and 2 are almost identical proteins, and *BIP3* is expressed at elevated levels in response to abiotic stress or ER stress agents (Koizumi, 1996; Martinez and Chrispeels, 2003). The overexpression of either *BIP1* or *BIP3* by transgenesis delays the release of bZIP28 and does not allow the complete deployment of bZIP28 to combat stress. As for BIP underexpression, double homozygous mutant lines of BIP1 and 2 are lethal in *Arabidopsis*, but hemizygous knockout lines have been produced (Maruyama et al., 2010). In hemizygous BIP knockout lines, bZIP28 is released from the ER even under unstressed conditions (Srivastava et al., 2013). Thus, the results involving the overexpression and underexpression of BIP in *Arabidopsis* support the dynamic equilibrium model and demonstrate the critical role that BIP plays in the retention and activation of bZIP28 in *Arabidopsis*. The model in plants has been further supported by the observation that overexpression of BIP in tobacco helps to alleviate ER stress responses (Leborgne-Castel et al., 1999). It was found that overexpression of a BIP transgene downregulated endogenous BIP mRNA levels and reduced the UPR.

However, detractors of the dynamic equilibrium model argue that BIP is present in millimolar quantities in the ER and that slight fluctuations in folding state of ER proteins would not be able to shift the equilibrium and compete BIP away from its binding to ER stress sensor/transducers (Credle et al., 2005). Therefore, another model for the release of ATF6 from BIP in animal systems has been evoked that does not involve dynamic competition. Instead this model proposes that the association is stable but can be disrupted by a signal from misfolded proteins. Several arguments favor a stability model (Shen et al., 2005). BIP appears to recognize ATF6 as an unfolded client protein in that mutations in the substrate-binding domain of human BIP (such as P495L) inhibited the binding of BIP to ATF6. Also mutations in the ATPase domain (such as T37G) prevented the dissociation of BIP from ATF6 by ATP even when these complexes were purified. However such complexes were dissociated very efficiently when ER stress was induced by dithiothreitol (DTT), though not by detergents *in vitro*.

### OTHER FACTORS THAT MAY REGULATE THE ACTIVATION OF bZIP28

The ER is a calcium-rich, oxidizing environment and imbalances in energy charge levels, redox conditions or interaction with DNAJ proteins could activate ER stress sensors. It has been observed in mammalian cells that the LD of ATF6 forms inter- and intramolecular disulfide bridges between its two conserved cysteine residues. In the absence of ER stress, ATF6 is found as monomer, dimer and oligomers in the ER (Nadanaka et al., 2007; Sato et al., 2011). Upon ER stress, due to the reduction of disulfide bridges, ATF6 is thought to depolymerize and to exit from the ER.

The ER has evolved specific posttranslational modifications and quality control mechanisms to prepare proteins for the extracellular environment. These modifications dramatically enhance the stability of secreted proteins. A major posttranslational modification of ER-synthesized proteins is disulfide bridge formation, which is catalyzed by the family of protein disulfide isomerases (PDIs; Ellgaard, 2004; Banhegyi et al., 2007; Andeme Ondzighi et al., 2008; Feige and Hendershot, 2011). PDI is the founding member of the ER PID family. Treatment of animal and plant cells with DTT results in ER stress due to the disturbance in the redox balance of the cells (Frand and Kaiser, 1998; Liu et al., 2007). The LD of ATF6 was found to associate physically with PDI under unstressed conditions implicating its role in imparting stability to ATF6 in the ER (Sato et al., 2011). Similar associations may also be expected with bZIP28 that might influence its function.

Hong et al. (2004) had shown that the glycosylation status of ATF6 is important in its interaction with the chaperone calreticulin. Under ER stress conditions, ATF6 is undergylcosylated, a condition which fails to promote its association with calreticulin and its retention in the ER. Liu et al. (2007) showed that the bZIP28 is glycosylated and, therefore, its glycosylation status may influence its interactions with ER chaperones and its retention in the ER.

DnaJ/Hsp40 (heat shock protein 40) proteins are important factors in chaperoning and protein folding primarily by stimulating the ATPase activity of Hsp70 proteins, which stabilizes the interaction of these chaperones with their substrate proteins (Shen and Hendershot, 2005). Six ER localized DNAJ proteins that have been identified and are referred to as ERDdj1–6 (Jin et al., 2009). One of these, ERdj-3, is a soluble lumenal DNAJ family member. It is known to bind to BIP chaperone complexes in the ER and associates with a number of other unfolded proteins that are BIP substrates (Jin et al., 2008, 2009). An ERdj-3 mutant that does not bind to BIP still retains its ability to bind to unfolded proteins directly. BIP assists in the release of ERdj-3 from its substrate. The mutants of BIP that do not allow the release of ERdj-3 disrupt these association–dissociation processes (Awad et al., 2008; Jin et al., 2008). Erdj-3 is therefore a candidate for binding to the LD of ER membrane associated bZIP transcription factors such as bZIP28 and to contribute to their activation.

### EXIT OF bZIP28 FROM THE ER AND FURTHER ORGANELLE TRANSLOCATION

The release of bZIP28 from BIP corresponds closely with its exit from the ER, but its release is not dependent on the ability of bZIP28 to traffick from organelle to organelle. As described above, in animal systems, it is thought that BIP binding retains ATF6 in the ER under unstressed conditions, putatively by blocking ATF6’s GLSs, preventing its transport through the secretory pathway (Shen et al., 2002). BIP’s release unmasks the two GLSs, GLS1, and GLS2 on ATF6. GLS1 binds to BIP while GLS2 is inactive. On dissociation of BIP, GLS2 directs ATF6 to the Golgi. Sequences similar to the GLSs in ATF6 have not been detected in bZIP28. Dissociation of BIP from bZIP28 only enables it to exit from the ER. Further organelle-to-organelle movement of bZIP28 is governed by other factors.

The mechanism of transport of proteins from the ER to the Golgi in plant cells is not completely resolved. It is unclear whether plants utilize intermediate compartments in the movement of ER cargo to the Golgi (Yang et al., 2005). The exit of cargo from the ER to the Golgi in yeast and animal cells involves COPII vesicles, but COPII vesicles have yet to be visualized convincingly in plant cells. However, mutations that affect the COPII system in plants disrupt protein transport from the ER to the Golgi (Marti et al., 2010). Some of the factors involved in the initiation of COPII vesicle assembly are a GTPase, Sar1, and Sec12, a guanine nucleotide exchange factor (Miller and Barlowe, 2010; Russell and Stagg, 2010). bZIP28 has been shown to interact with Sar1b and Sec12, and this association appears to play an important role in the translocation of bZIP28 from ER to the Golgi (Srivastava et al., 2012). Sar1b is one of the several plant Sar1 forms identified in *Arabidopsis* (Hanton et al., 2008). Sar1 can further recruit Sec23/24, the inner COPII vesicle components (Kuehn et al., 1998; Aridor et al., 2001; Bi et al., 2002).

A basal level of interaction between Sar1b and Sec12 with bZIP28 is seen even under unstressed conditions but stress treatment enhances this interaction several fold. This basal level of interaction between bZIP28 and COPII components is apparently not sufficient to initiate the transport of bZIP28 to the Golgi. Under stress conditions, a threshold level of interaction between bZIP28 and Sar1 is apparently met, and bZIP28 is mobilized (Srivastava et al., 2012). The interaction of bZIP28 with Sar1 requires the presence of dibasic residues on the cytosolic side of bZIP28 near the TMD (**Figure 2**). Two neighboring KK motifs at this location have a combined role in Sar1 binding to bZIP28, because substitution of charged residues to this pair of motifs results in a loss of Sar1b binding (Srivastava et al., 2012). It is believed that a charged pocket is created as a result of the paired lysines existing in close proximity to each other (Giraudo and Maccioni, 2003). ATF6 has also been shown to translocate to the Golgi with the aid of COPII vesicles (Schindler and Schekman, 2009).

Once bZIP28 exits the ER and moves into the Golgi, S2P proteolytically processes and releases the cytoplasmic-facing components of bZIP28 from the Golgi. S2P is an intramembrane metalloprotease (Feng et al., 2007) involved in regulated intramembrane proteolysis (RIP). The targets for intramembrane proteases are TMDs, and important residues in the TMDs of substrate proteins for a class of intramembrane proteases called rhomboids have been identified (Freeman, 2004). The substrates for S2P in mammalian systems are TMDs with a mid-domain helix-breaking residue and hydrophilic residues at the membrane boundary that are thought to provide a water channel for the intramembrane hydrolysis reaction (Urban and Freeman, 2003). The helix-breaking residues are thought to destabilize the TMD α-helix, causing a locally disordered conformation of the TMD and providing access of the protease to the substrate. Some of these residues are also found in the TMD of bZIP28, and a G329A mutation introduced into the middle of bZIP28’s TMD demonstrated the importance of a helix-breaking G residue for proteolysis by S2P. The mutation resulted in a loss of bZIP28 processing, leading to its retention in the Golgi and a block in its translocation to the nucleus (Srivastava et al., 2012).

A hydrophilic VASI sequence at the cytosolic face of the TMD was hypothesized to be a channel permitting entry of water into the membrane interior for hydrolysis of the bond cleaved by rhomboid proteases (Urban and Freeman, 2003). To determine if the VASI sequence in bZIP28 was likewise required for proteolysis, it was substituted by a sequence to reduce the hydrophilicity of the region at the cytosolic boundary of bZIP28’s TMD. These mutations had no effect on the proteolysis and movement of bZIP28 (Srivastava et al., 2012). Hence, *Arabidopsis* S2P may utilize a different mechanism for water entry to catalyze the intramembrane hydrolysis of bZIP28.

## CONCLUSION AND FUTURE INSIGHTS

The sensing of ER stress involves the association or dissociation of the ER chaperone, BIP, from bZIP28. bZIP28 is retained in the ER during unstressed conditions by its interaction with BIP. Likewise, the mobilization of bZIP28 in response to stress involves its dissociation from the BIP and its association with COPII factors on its cytoplasmic face. Further organellar movement of bZIP28 is guided by residues in the TMD. Other unknown factors may also have a role in the activation and movement of bZIP28 and need to be explored.

## Conflict of Interest Statement

The authors declare that the research was conducted in the absence of any commercial or financial relationships that could be construed as a potential conflict of interest.
